# Remarks on Revaccination

**Published:** 1839-03-30

**Authors:** F. Campbell Stewart

**Affiliations:** Williamsburg, (Va.)


### REMARKS ON REVACCINATION.

By F. C. Stewart, M. D.

To the Editors of the Medical Examiner.

Gentlemen,—The great and vital importance of the subject will, I hope, be a sufficient excuse for my requesting the insertion in your valuable journal of a few facts and remarks on the necessity for revaccination, and the present value of our vaccine matter as a prophylactic against small-pox.

The history of vaccination, its introduction by Jenner, and the immense benefit that it has proved to mankind, are matters so well known, that it would be more than superfluous to occupy your space in detailing facts which have become a part of history. It is to the present condition of things, with the absolute necessity for revaccination, and the apparent failure of the present vaccine lymph to insure immunity from variolous contagion, that I would call the attention of the profession.

The venerable discoverer of vaccine himself recommended revaccination; and, since his time, the profession has been always divided as to the necessity of the measure. By a series of experiments, however, made on a large scale during the years 1831–2, on the Prussian and Wurtembergian armies, by order of their respective governments, it was proved most conclusively, that after a certain time, varying in individuals, matter which had been introduced into the system, ceased to retain its protective power.

In the third corps of the Prussian army, 6,020 individuals were revaccinated. Out of this number, 2,354 (more than 39 per cent.) presented, at the usual time, the true vaccine vesicle. In the eighth corps of the same army, 925 out of 2,784—and in 1832, 3,942 soldiers having been revaccinated, 1,594 presented the true vesicle. The average proportion here, then, is about one-third of those in whom the vaccine took for the second time. When it is borne in mind that no soldier is ever admitted into the Prussian army who does not produce a certificate of vaccination, and that the above experiments were performed with the greatest care, there can be no doubt of the necessity of revaccination in the cases in which it was practised. But we have further the authority of Dr. Heim, of Ludwigsburg, who published an account of the experiments conducted in the Wurtembergian army,—the effects of which were, that from 30 to 40 per cent., or about one-third of those revaccinated, were found to be susceptible of a second impression,—a result in perfect accordance with that obtained in the Prussian army. Dr. Heim was of opinion that seventeen years is the extreme period for which the anti-variolous power of the vaccine matter may endure.

M. Dezeimeris has lately published on the subject of revaccination; and it is from a review of his work, to be found in the Gazette Médicale, of Paris, for 1838, No. 53, and page 829, that I shall make some extracts, which will go to show—1st, the actual original value of the vaccine matter; and, 2dly, the necessity for revaccination.

As he considers them the most authentic, M. Dezeimeris has selected from the documents furnished by Denmark, and particularly those of its capital, the following account of small-pox, and its ravages, both preceding and subsequent to the discovery of Jenner, and the degree of power possessed by vaccine during the first years of its use, to prevent small-pox. From 1749 to 1808, there died of the disease at Copenhagen, between the years—

| | | |
|---|---|---|
| 1749 and 1758, | . . | 2,991 persons. |
| 1759 and 1768, | . . | 2,068 " |
| 1769 and 1778, | . . | 2,224 " |
| 1779 and 1788, | . . | 2,028 " |
| 1789 and 1798, | . . | 2,920 " |
| 1799 and 1808, | . . | 724 " |

From the year 1800 to 1804, no case of small-pox was reported as having occurred in a person who had been vaccinated. In 1804 two cases occurred, but both were of varioloid. In 1805 five persons died, and in 1806 three, also of varioloid. In 1808 forty-six variolous cases proved fatal, thirteen of which were varioloid.

In 1819, cases of varioloid, and even of the genuine small-pox, began to appear throughout Denmark, in a great number of those persons who had undergone vaccination; and in 1823, so great a number were attacked, that it became necessary to establish a small-pox hospital for the poorer classes at Copenhagen. The charge of this hospital was given to Dr. Moehl, who published three reports of three successive invasions of small-pox from 1824 to 1827. Of 412 patients who were received into the hospital during the first thirteen months, 257 had been vaccinated—58 had already had the small-pox—97 had neither been vaccinated nor had the small-pox—(a large proportion of these last being of adult age at the time of Jenner's discovery, had thought the precaution unnecessary.)

It will be useful to state the ages of those of the variolous patients who had already been vaccinated, as it will show what length of time had elapsed since their vaccination: 24 were under 7

years, 42 between 7 and 11, and 191 between 12 and 23. This statement shows that nine-tenths of them had been vaccinated more than ten years, supposing that they availed themselves of the advantages to be derived from the discovery at the time that it was made. 40 out of the 412 cases proved fatal, of whom 3 only were of the 257 who had been vaccinated.

From 1825 to 1827, out of 623 cases of small-pox and varioloid, 438 were persons who had undergone vaccination—of whom 26 had a small-pox differing in no way from the disease occurring amongst those who were not at all protected, and two of them died. Since the year 1825, recourse has been had to revaccination.

M. Dezeimeris further goes on to show from his documents, that out of an increased number of patients admitted into the Copenhagen hospital, not one under the age of 14 was affected with the true small-pox—not a single fatal case occurred in a subject under 23 years, and not one case of variola in a revaccinated patient.

At the present time the disease seems to be on the increase in Europe, where, in consequence of the supposed or real failure of the vaccine matter to insure its desired end, great uneasiness is felt both by the profession and community. At a late meeting of the Royal Medical and Chirurgical Society of London, Dr. Gregory, physician to the small-pox hospital of that city, stated, that in the middle of November, 1837, there had been a sudden and marked increase in the number of patients admitted into the hospital, which increase had been gradually continuing. From the 1st of January, 1838, up to the time of making his report, (in December last,) 681 patients had been admitted, 281 of whom had been previously vaccinated.

The above well authenticated facts alone are quite sufficient to show the necessity for a general revaccination in Europe! Unfortunately, in this country we have no means of investigating the subject, so as to enable us to form any very correct conclusions as to the necessity for it with us. But, if requisite in Europe, we must also stand in need of additional and more sure protection from so fearful a malady.

With regard to the deterioration in quality of the vaccine lymph now in use, much might be said, and many instances cited of its entire failure to produce its characteristic vesicle. Indeed there are but few practitioners who have not been repeatedly dissatisfied with the result of their vaccinations, and many have noticed its gradual but continued alteration from the time of its introduction to the present day. The vesicle of the present matter differs materially from that described by Jenner and his cotemporaries. The scar even does not present the same characterizing and *genuine* appearance. It is not my intention now to examine into the cause of the diminished efficacy of our matter; nor to trace the changes and modifications which it has probably undergone in passing through such numbers of systems as have availed themselves of its wonderful powers. My sole object is to excite attention and draw inquiry to the subject, hoping that some plan may be devised for ascertaining with accuracy, by experiment, the condition of such individuals as have already been vaccinated, and their susceptibility of a second vaccine impression, with a more close inquiry for a new source from whence we may obtain a fresh supply of uncontaminated matter.

Hoping that the above remarks may not be deemed superfluous, and that they may have the effect of promoting investigation

I remain, gentlemen, very respectfully yours,

F. Campbell Stewart.

*Williamsburg*, (*Va.*) February 25th, 1839.

---

To the Editors of the Medical Examiner.

Gentlemen,—In your tenth number a case is mentioned to prove the antiquity of Lithotrity. In the first volume of the London Medical and Physical Journal, you will find a statement by Col. Martin, an officer in the British East India service, of his having destroyed a calculus in his bladder by a file.

I am, respectfully, James Mease.

*Philadelphia*, March 25th, 1839.

